# The Life of Sir Astley Cooper, Bart., Interspersed with Sketches from His Note-Books of Distinguished Contemporary Characters

**Published:** 1843-07

**Authors:** 


### Art. VII.

*The Life of Sir Astley Cooper, Bart., interspersed with Sketches from his Note-books of distinguished contemporary characters.* By Bransby B. Cooper, Esq. f.r.s. In Two Volumes.—*London*, 1843. 8vo, pp. 448, 480.

When we learned that Mr. Bransby Cooper had in the press a memoir of his uncle, Sir Astley, we naturally expected that the history would have reference chiefly to that profession of which the subject was so bright an ornament, and the author a distinguished member; nor was it without disappointment that we discovered, on taking up the volumes, that they contained simply the life of Sir Astley Cooper as an individual. Biography of this kind affords, for the most part, little that is interesting or instructive. Men may be very illustrious in a professional or public capacity, and yet differ very little from ordinary mortals in the details of their private life. They are born like others; they live by the exercise of similar functions; they have their peculiarities of disposition and of temper; their personal attachments and aversions; they die, and are remembered by the world only through the enduring influence of the master mind, which has impressed its own character on some particular department of human knowledge or human activity.

The only individuals whose private history is possessed of much interest are those who have been involved in striking personal adventures. However impressive may be the scientific life of a Newton or a Kepler, the political history of a Richelieu or a Chatham, or the military career of a Napoleon or a Wellington, the private life of any one of these individuals will contain very little more than that of the most commonplace man in the world, and will be far inferior in interest to that of a Turpin or a Vidocq, or any one, good or bad, who has passed through extraordinary vicissitudes of fortune, or surmounted extraordinary personal dangers and difficulties.

Our author tells us in his introduction, that "there is no doubt that Sir Astley Cooper intended that whatever biographical memoirs of him might be published, should comprise an analysis of his professional writings, an account of the circumstances under which they were produced, their peculiar merits, and a comparison of them with the existing state of knowledge at the time of their appearance; that, in short, they should afford a complete view of him, both as the surgeon and the author." Considering, however, that such a biography would have been a sealed book to all but professional readers, and conceiving that the public would be gratified by "an insight into the habits and pursuits of a man, who for many years served them extensively in his professional capacity, and in whom they always exhibited the greatest interest," Mr. B. Cooper determined on publishing, at present, a merely personal memoir of Sir Astley, and deferring to a future opportunity the history of his strictly professional life. We regret extremely that Mr. B. Cooper has acted on this resolution. Sir A. Cooper, we know, was much and worthily beloved by his friends, but we apprehend that the "interest" taken in him by the public, arose simply from their admiration of his character as a surgeon. Take him out of his profession, and his history is merely that of a worthy and kind-hearted man, clever, and somewhat given to waggery, endowed with a shrewd eye to his own interest, and a benevolent disposition to promote the welfare of others. This is not a very remarkable character, nor one requiring two octavos for its delineation. We do not think the memoir before us will be found to possess as much general interest as the author imagines; and, unhappily, it lacks that which would have made it in the highest degree interesting to the medical reader, viz. a critical retrospect of Sir A. Cooper's professional life; a comparison of his surgical character and labours with those of Scarpa, Dupuytren, and other illustrious surgeons of his day, and a comprehensive view of the progress of the art throughout Europe during the last half century. The name of Astley Cooper, however, has been so long familiar to the world, and his great talents and well-known *bonhomie* have so endeared his memory to his own profession, that we have no doubt even the memoir before us will be perused by many of that profession with considerable interest. It has moreover one curious feature, which, although anomalous, and derogatory from its character as a classical biography, renders it entertaining, and in some respects valuable, namely that of giving a sketch of the lives or characters of all the persons with whom Sir Astley was thrown into any intimate relation, and many of whom were necessarily persons of distinction.

Under ordinary circumstances, purely biographical works do not come within the range of criticism prescribed to this Journal. In the present instance, however, the great professional eminence of the individual who is the subject of the memoir, induces us to deviate from our usage, and leads us further, to present our readers with an outline of the personal history of Sir Astley Cooper, for the materials of which we are almost entirely indebted to the volumes before us.

Astley Paston Cooper was born at Brooke, in Norfolk, on the 23d of August, 1768. He was the fourth son of the Rev. Dr. Samuel Cooper, who then held the rectory of Brooke, and his wife, Maria Susanna, eldest daughter of James Bransby, Esq. of Shotisham. In the year 1781, Dr. Cooper was appointed to the perpetual curacy of Great Yarmouth, and, on obtaining that preferment, left Brooke, where he had resided for thirteen years, and where he appears to have been much beloved by his parishioners. His affluence and hospitality made his acquaintance much sought for, and among his visitors were some of the most celebrated literary and political characters of the day. He possessed, in an eminent degree, the feelings and habits of a gentleman, though his disposition was tinctured with austerity, and was not altogether free of the dogmatism into which men much courted in provincial society are so apt to fall. He was the author of numerous publications, on religious and political subjects. He died at Great Yarmouth, in January, 1800, in the sixtieth year of his age.

Maria Susanna, the mother of Sir Astley Cooper, was born at Shotisham, in the year 1737. Her father, James Bransby, Esq. was descended from an ancient family in Yorkshire, a scion of which afterwards settled at Harleston, in Norfolk. Her mother, Anna Maria Paston, was the daughter of James Paston Esq. of Harleston, a first cousin of the Earl of Yarmouth. She appears to have been a lady of rare endowments both of mind and heart. She published several works, chiefly novels, having a moral or religious tendency, which enjoyed a high reputation in their day. She survived her husband some years, and died in July, 1807.

In conformity with a very absurd and pernicious custom then prevalent among the more respectable families of Norfolk. the infant Astley Cooper was transferred to the care of a foster-nurse, Mrs. Love, the wife of a substantial farmer, one of Dr. Cooper's parishioners.

As soon as he was of an age to receive instruction, his mother undertook the care of his general education in common with that of her other children, and his father subsequently initiated him into the rudiments of classical learning. As is well known, however, he never attained any great proficiency as a scholar, his turn of mind being entirely different from that which leads to excellence in philological pursuits. His only other preceptor, at this period, was Mr. Larke, the master of the village school, who used to attend at Brooke Hall to instruct Dr. Cooper's children in writing, ciphering, and mathematics. Among all this gentleman's pupils, none seems to have done him less credit than young Astley. Mr. Lark, it appears, laboured under so great a susceptibility of external impressions in the epigastric region, that if he received a slight blow, or was merely touched in that part, he was for some time deprived of respiration. Of this infirmity our young Pickle did not omit to avail himself on certain occasions, where the state of his own studies rendered a suspension of his tutor's faculties desirable.

He grew into a lively and daring boy, always in mischief, yet so open-hearted and good-humoured, that no one could retain their anger against him long. He delighted in all sorts of extravagant and dangerous enterprises; he would ride the most vicious horses without a bridle, and drive the cows out of the fields mounted on the back of a bull; and, on one occasion, he fractured his collar-bone in an attempt to leap an ass over a recumbent cow, who was so disobliging as to get up in the midst of the exploit, and roll him and his Bucephalus on the field.

A short time previous to Dr. Cooper's removal from Brooke, an accident occurred which afforded striking indications of the presence of mind and aptitude for practical surgery by which Astley Cooper was afterwards so much distinguished. A son of his foster-mother, Mrs. Love, somewhat older than himself, having been sent to convey some coals to the house of the vicar, fell down by some accident in front of the cart, a wheel of which, passing over his thigh, caused, among other injuries, a laceration of its principal artery. The boy was carried home almost exhausted with hemorrhage, and all was alarm and confusion, when Astley Cooper, who had heard of the accident, arrived, and with admirable presence of mind bound his pocket-handkerchief so tightly around the limb above the wound, as to arrest the bleeding. In this manner the life of the patient was prolonged till the arrival of the surgeon who had been sent for from Loddon. Sir Astley Cooper used to relate that this accident, which he always regarded as a remarkable event in his history, first bent his thoughts towards the profession of surgery.

On his arrival at Yarmouth, Astley Cooper continued as great a madcap as ever, and some of his pranks evince a keen perception of fun and considerable talent in the invention of ludicrous positions. The following eccentric contribution to meteorology is amusing enough :

"Having taken two pillows from his mother's bed, he carried them up to the spire of Yarmouth church, at a time when the wind was blowing from the north-east, and as soon as he had ascended as high as he could, he ripped them open, and shaking out their contents, dispersed them in the air. The feathers were carried away by the wind, and fell far and wide over the surface of the market-place, to the great astonishment of a large number of persons assembled there. The timid looked upon it as a phenomenon predictive of some calamity, the inquisitive formed a thousand conjectures, while some, curious in natural history, actually accounted for it by a gale of wind in the north blowing wild-fowl feathers from the island of St. Paul's. It was not long, however, before the difficulty was cleared up in the doctor's house, where it at first gave rise to anything but those expressions of amusement which the explanation, when circulated through the town, is reported to have excited." (pp. 73-4.)

On another occasion he secreted himself in the church, and obliged the Rev. Dr. Cooper with an exact echo of his voice while reading the marriage service. The doctor gravely observed that he had never perceived an echo in that place before; while the clerk, who had a shrewd guess at the real nature of the phenomenon, could with difficulty restrain his laughter, though filled with alarm lest the trick should be discovered.

But amid these frolics Astley Cooper now began to feel dissatisfied with his mode of life, and conceived a growing desire of occupation, independence, and distinction. The bent towards surgery, which he had acquired from the circumstance already related, was greatly increased by the conversation of his uncle, Mr. William Cooper, who occasionally visited his father, and was at that time senior surgeon to Guy's Hospital. Their intercourse affording mutual pleasure, it was finally determined that Astley should embrace the profession of surgery, and he was accordingly articled as pupil to his uncle.

Our student set off for London in August 1784, leaving his family in a state of solicitude as to his future conduct, which was naturally engendered by his previous unsettled habits, and neglect of the more serious duties of life. It being inconvenient to Mr. W. Cooper to receive his nephew into his own house, it was determined that he should reside at that of Mr. Cline, who then took a few pupils to board with him. This arrangement turned out highly advantageous to Astley Cooper; for his uncle, though warmly interested in his success, and entertaining a high opinion of his good qualities and abilities, had a certain roughness of manner, and strict notions of discipline which might have ill accorded with the impetuous and ungovernable disposition of his pupil.

For a short time, there seemed some reason to fear that the apprehensions of Astley Cooper's family with respect to his conduct would be realized. His handsome person and engaging manners rendered him a general favorite in society, and his lively temperament, as yet uncontrolled by addiction to any serious pursuit, led him into a gay and dissipated course of life; while the love of adventure, which still adhered to him, occasionally urged him into extravagances of conduct like those which had characterized his earlier years. His connexion with Mr. Cline, also, though highly desirable in a professional point of view, was very much the reverse in some other respects. The political principles of that gentleman brought him into the closest intimacy with Horne Tooke, Thelwall, and other leaders of the Jacobin party in England. The ardent and undisciplined mind of Astley Cooper made him an apt pupil in such a school, and he became deeply tinctured with political fanaticism. Fortunately, however, a growing fondness for his profession soon engaged him better than in the pursuit of chimeras. At Christmas 1784, he was transferred, as an articled pupil, from Mr. Cooper to Mr. Cline, and about this time he seems to have laid aside his idleness, and to have devoted himself earnestly to the study of surgery. So great was his diligence that, by the spring of the following year, he had become as distinguished for industry as he had before been notorious for wasting his time, and had, in particular, attained a degree of proficiency in anatomy far exceeding that of any other pupil of his own standing at the hospital.

It is uncertain whether, at the conclusion of his first winter session, he remained in London, or paid a visit to his family at Yarmouth, though the latter is more probable. In October 1785, Astley Cooper resumed his studies with renewed ardour. He was led to the particular cultivation of anatomy, both as the only solid basis of medical and surgical knowledge, and as the chief means of professional advancement for a young man in the position in which he then stood. The demonstratorship of anatomy at the school of a large metropolitan hospital has always been deemed important as a first step in the career of an operative surgeon, and was considerably more so at that time than it is at present, from the smaller number of those capable of fulfilling its duties. The office of demonstrator at Guy's was at that time filled by Mr. afterwards Dr. Haighton, whose want of affability seems to have rendered him unpopular among the students. Astley Cooper had already so distinguished himself by his superior knowledge of anatomy as to be generally referred to by his fellow-students in their difficulties; and this, together with his connexion with Mr. Cline, caused him to be regarded as a sort of second demonstrator, while his kindly disposition and frank address made him a universal favorite among the pupils. These circumstances encouraged him to look forward to the place of demonstrator in the event of its becoming vacant. Having rendered himself an efficient anatomist, he began to feel the advantages of accompanying Mr. Cline in his visits to the Hospital. He watched the progress of the cases with a scrutinizing curiosity, carefully examining the nescropic appearances in such as were fatal. He took notes of Mr. Cline's cases, and soon became remarkable for his quickness in seizing upon their leading points.

During the first winter of his studies, Astley Cooper had become a member of the Physical Society, the oldest and one of the most valuable institutions of its kind at that time existing in London. He seems, however, to have been a very negligent member; and we learn that, in the course of the session, he was fined for absence no less than fifteen times. In the second session of his studies, however, we find him absent only once. Indeed, his mind seems now to have become thoroughly impressed with the importance of his studies, and imbued with the love of them; and his conduct, instead of exciting alarm lest he should become an incompetent member of his profession, called forth feelings of pride and satisfaction from his friends at home, and congratulations from his teachers and acquaintance at the hospital.

In the summer of 1786 he again visited Yarmouth, where, instead of wasting his time in his former vagaries, he did not even spend it in mere recreation, but engaged himself much in the surgery of Mr. Francis Turner, an intelligent general practitioner, with whom he was intimate, with a view of acquiring a knowledge of pharmacy. Truth, however, obliges us to observe, that he must either have profited little by his experience at Mr. Turner's, or forgotten in after-life much of what he had acquired; for Sir Astley was notoriously deficient in pharmaceutical knowledge. He also, during this visit to Yarmouth, conversed much with the well-known Dr. Aikin, and with his friend Mr. Holland, then a highly intelligent medical student, the father of the celebrated author of the Medical Notes and Observations.

On his return to town he renewed his studies with increased ardour. If he had imbibed from Mr. Cline some tenets which he would have been better without, he derived, from the same source, a predilection which had a very favorable influence on his professional character; and he became, like his preceptor, an enthusiastic admirer of John Hunter, a course of whose lectures he attended. The obscurity of expression which rendered the writings and discourses of that great man so difficult to understand, did not deter him from unravelling his meaning by earnest attention, while, by discussing the subject of each day's lecture with his friend Mr. Holland on his way home, as well as by private experimental inquiry, he succeeded in fixing Hunter's principles in his mind, and became fully convinced of their truth and importance.

About this time, Astley Cooper was near falling a victim to malignant fever, contracted by a visit he paid, at the request of his father, to a person named Gregson, who was a prisoner in Newgate, and whose history Mr. B. Cooper has thought sufficiently interesting to be recorded. We think otherwise: suffice it therefore to say that he was hanged. Astley Cooper's illness proved very dangerous; but he recovered under the care of Mr. Cline and his family. It was not, however, until he had for some time breathed the air of his native county, that he was completely restored to health.

In the winter of 1787, he visited Edinburgh, carrying with him letters of introduction to the most eminent medical and scientific men of that city. Having presented his letters, he immediately applied himself to the objects of his journey; and, for the convenience of doing so, hired a lodging close to the principal scene of his studies. Neither his abode nor his style of living appear to have been of a very sumptuous character, judging from a note of his own relating to this period:

"August 30th. Walked out to the college, and to Bristow Street, and saw the infirmary. Saw my lodging, No. 5, in Bristow Street; walked up into my room, where I spent six shillings and sixpence per week in lodging; dining in Buccleugh Place with Mrs. Mackintosh, at one shilling per diem."

In this part of the memoir Mr. B. Cooper gives various anecdotes of the principal men who figured in Edinburgh at that time, which can interest only those who were acquainted with the place and the period. As a specimen of the kind of notes which Astley Cooper was wont to make, we subjoin a few written after he left Edinburgh, and retrospective of some of the characters he there met with. They exhibit some vivacity, but otherwise nothing remarkable.

"Adam Smith was good natured, simple-minded, unaffected, and fond of young people. Mackenzie I saw little of. Gregory's lectures on clinical medicine were admirable; yet he thought most highly of his physiology, on which he enlarged in his evening lectures on therapeutics. Having on one occasion been confined to my room by illness, I expressed my regret to Dr. Gregory at losing his clinical reports, but he said, 'Sir, that does not signify; but you have lost my therapeutics.' Black said, 'Sir, you will speak to me after lecture if you do not understand anything. Have you fixed upon a tailor or a shoemaker? I can recommend you to one and the other:' but seeing he might carry his furnaces in his shoes, and that his coat was probably like that worn by Noah in the ark, I thankfully declined. Lord Meadowbank was a sharp man, something like Wollaston. Charles Hope was a man of reading, a gentleman, and dignified, and very eloquent. Old M—— grunted like a pig. He was a tolerable lecturer, possessed a full knowledge of his subject, had much sagacity in practice, was laudably zealous, but was much given to self and to the abuse of others. I gave him two instruments, Cline's gorget, and an instrument for scratching the capsule of the lens, and the next day he said, 'Gentlemen, Mr. Cooper has given me two instruments, one for scratching the capsule of the lens, which *may be* useful; the other, a cutting gorget, and it is curious I myself invented this very instrument twenty years ago.' Fyfe I attended, and learned much from him. He was a horrid lecturer, but an industrious worthy man, and a good practical anatomist. His lecture was, 'I say—eh, eh, eh, gentlemen; eh, eh, eh, gentlemen—I say,' &c.; whilst the tallow from a naked candle he held in his hand ran over the back of it and over his clothes: but his drawings and dissections were well made, and very useful.

"I was glad I went to Edinburgh, because I learned that distance enhances the character of men beyond their deserts. Cullen, and Black, and Dugald Stewart, however, were great men, and being near them did not diminish the importance I had been led to attach to them from their public character. Dugald Stewart was beyond my power of appreciation,—metaphysics were foreign to my mind, which was never captivated by speculation;—but Dr. Black's lectures were clear, and I knew enough of the subjects he treated upon to understand them. Never shall I forget the veneration with which I viewed Cullen; he was then an old man: physic may have much improved since his time, but if Hippocrates was its father, Cullen was its favoured son." (Vol. I, pp. 170-2.)

While in Edinburgh, Astley Cooper became a member of the Royal Medical Society, and so distinguished himself in its discussions that he was offered the presidency in the event of his return to Edinburgh, which, however, never took place. He was also a member of the Speculative Society, and read before it a paper in support of the Berkeleian doctrine of the non-existence of matter; but this he probably maintained only for the sake of argument, as the society in question was considerably more given to ratiocination than to the establishment of fact.

At the period of Astley Cooper's visit to Edinburgh, his comparison of the state of surgery in that city with the state of the same art in London could not have been very favorable to the former; he nevertheless formed a high estimate of the general state of medical education in Edinburgh, and attributed much of his well-known facility of concentrating his whole knowledge and experience on each case that came before him to the correct order and comprehensiveness of the Edinburgh course of study.

At the conclusion of the session, he made a tour to the Highlands which extended to the Western Isles; after which, beginning to be tired of leisure, and not a little reduced in his pecuniary resources by an inconsiderately expensive entertainment which he had given to his friends previously to leaving Edinburgh, he bent his steps homeward, and arrived in London towards the end of Autumn 1788, improved in health, professional knowledge, and general information. In his memoranda are the following allusions to this period :

"When I returned to London, I found that I had learned much during my absence, and in seeking the sources from which I had derived most information, Drs. Ash, Gregory, and Fyfe seemed specially to claim my gratitude. Big with my own importance, I became presumptuous, but was soon taken down by Newell, afterwards of Cheltenham ; Shrapnell of Berkely : and others in the Physical Society." (Vol. i, p. 181.)

At this time he formed an intimacy with Mr. Colman, who became a pupil of Cline, and this intimacy ripened into a close friendship, which was cemented by the mutual assistance rendered in their studies, and the high distinction subsequently attained by each in his particular department. Their friendship continued uninterrupted till Mr. Colman's death. Sir A. Cooper has left among his papers a brief biography of Mr. Colman, from which some extracts are given in the work before us. These passages are very neatly and appropriately written, and convey a more favorable idea of Sir Astley's literary style than we should have formed from his published works.

From the winter 1788 until the year 1791, no record has been preserved of any particular events in the life of Sir Astley Cooper ; but, at some time during this period, he was appointed demonstrator of anatomy at St. Thomas's Hospital, in the place of Mr. Haighton, who had resigned. He appears to have been fully occupied with the duties of this office and with his professional studies till the year 1791. At this time his great proficiency in his profession, the connexions he had formed with eminent men, his high popularity with the students, and his well-known industry and energy, led Mr. Cline to perceive the advantages that would accrue to the school as well as to his pupil, from associating the latter with himself in the anatomical lectures. It was accordingly arranged that Astley Cooper should give a part of the lectures and demonstrations, Mr. Cline promising him the sum of £120 per annum, to be increased £20 annually, until he gave one half of the lectures, when the proceeds were to be equally divided. It had been customary in these lectures, to unite the subjects of anatomy and surgery, but Astley Cooper, who had for some time seen the necessity of devoting to each a separate course, took the present opportunity of making a proposal to that effect, which, however, at first met with opposition even from his friend and perceptor Mr. Cline. But at last he carried his point, and it was agreed that he should himself take the surgical department, and deliver his lectures in the evening at St. Thomas's hospital. There cannot be a stronger proof of the estimation in which he was held than that so young a man, and still merely a pupil, should have been able to bring about so important a change in an institution by no means unduly addicted to innovation. Mr. Cline had a motive in addition to his high opinion of his pupil, for wishing to advance him rapidly in his profession. Astley Cooper was under a matrimonial engagement to Miss Cock, the daughter of a friend and family connexion of Mr. Cline. Her father had made a considerable fortune as a Hamburg merchant, and having for some years retired from business, had taken up his abode at Tottenham. The marriage, which had been deferred by desire of the parents of both parties, till the termination of Cooper's pupilage, was to have been celebrated on the 21st of November, 1791, but was postponed on account of the death of Mr. Cock, which happened on that very day. Within a year from that time, however, the wedding took place, but was conducted with perfect privacy. On the evening of the same day on which the ceremony was performed, Mr. Cooper met his class as usual, delivering his lecture with his wonted ease and freedom of manner, and the pupils dispersed without the smallest suspicion of the change which had taken place in their preceptor's state. The new-married couple took up their abode at a house in Jefferies Square, which Mr. Cock, promising himself the happiness of seeing his daughter surrounded with every comfort, had, but a short time before his death, purchased and furnished for them.

Soon after the conclusion of the winter session, Mr. Cooper and his wife visited Paris, where the former anticipated much gratification and improvement, from a comparison of the French with the English surgery. His peculiar opinions, also, made him regard with interest the great political crisis of which Paris was then the scene. The frightful events of the revolution, which passed before his eyes, soon converted his feelings into those of disgust and horror, which were scarcely less excited by the fiendish expression and gestures of the democratic leaders in the National Assembly, than by the wholesale murders committed at their instigation. The alarm of his wife, and the peril and uncertainty of their situation, conspired with his own feelings to determine him immediately to return to England. A difficulty, however, of obtaining passports, protracted their stay till the middle of September, when they arrived in England in safety. The events which he had witnessed appear to have produced no change in the political tenets of Astley Cooper, but he had probably learned more from them than he liked to acknowledge, as his biographer tells us that he had always a repugnance to making any mention of the views he had entertained at this period. During his stay in Paris, he had attended the hospitals, and heard the surgical lectures of Desault and Chopart, but does not seem to have been greatly struck with the abilities of either of these celebrated surgeons. The latter he merely mentions in his notes as "a good kind of old woman, of little firmness of character." The notes, here published, in connexion with the Parisian expedition are so poor that they are not worth transcribing.

On his return to London, he began to give gratuitous advice at his own house early in the morning, which he seems to have done merely with a view of familiarising himself with disease in all its forms. At this period he was not very solicitious for private practice, his time being much occupied at the hospital. Fortunately, his circumstances in life emancipated him from all pecuniary anxieties, for, in addition to the income he derived from his lectures, he received, with his wife, fourteen thousand pounds.

His surgical lectures engaged much of his attention, and caused him considerable anxiety. He was disappointed to find that the number of pupils at his second course was less by twenty-five than it had been at his first. He had hitherto adopted the recently divulged opinions of John Hunter as the basis of his lectures; but it now struck him "that however just his first views had been in teaching surgery to his pupils upon the sound principles of John Hunter, still it was necessary, not only that his auditors should have a previous understanding of the meaning of John Hunter, but that they should have advanced to a considerable extent in the general knowledge of surgery, in order for them to comprehend the application of his theory to their practice. He therefore determined to change his plan; nor had he to go far a field to discover another which promised to be not only popular in itself, but more readily to secure the ultimate object he had in view, of so combining the doctrines of Hunter with the science of surgery, that they only should regulate its practice. For this purpose he selected the cases of disease, and the casualties admitted into the two hospitals, and, bringing such of them before the notice of the pupils as would illustrate the subject on which he was treating, he first pointed out to them the nature of the disease or accident, described the appropriate treatment, and afterwards inculcated the theoretical views which indicated it."

From this moment his class increased, and he became a highly popular lecturer on surgery. There was probably another reason why this change in the character of his lectures proved so beneficial, namely that he was not formed by nature for a framer or expositor of doctrines of any kind. His mind was of a strictly practical cast, and his chief endowments consisted in an extraordinary quickness and accuracy of perception, and a wonderfully retentive memory for facts.

At this time few of the surgeons in extensive practice had any confidence in the professional knowledge of John Hunter, whom they considered as a mere speculator; so unwilling is the world to do homage to living genius, while mere talent, if exercised in a popular direction, usually obtains more than its due meed of applause. But true genius has its bright reversion in after time, while mere cleverness enjoys only an ephemeral ascendancy.

In the year 1793, Mr. Cooper was appointed professor of anatomy to the College of Surgeons, and was reappointed in the two succeeding years. In November 1793, Mrs. Cooper was delivered of a female child which was christened Anna Maria. The bad health of this child proved a source of distress, and her death, in March 1794, a severe shock to both her parents. They afterwards adopted as their daughter, a child whom Mrs. Cooper accidentally met with at Hornsey.

In the year 1796, Astley Cooper accompanied Mr. Gawler to Hamburg, to act as medical attendant to that gentleman on the occasion of his duel with Lord Valentia. During the voyage homeward, they encountered a severe gale, and Astley Cooper afforded an example of the fact that the boldest have their moments of fear, and that the same mind which despises danger in one shape may cower before it in another. From the combined effects of terror and sea-sickness, he actually became delirious; and he was often afterwards heard to say " not the riches of the Indies should ever again induce me to make a longer voyage than from Dover to Calais." In all his subsequent visits to the continent, he invariably went by way of Calais.

Towards the end of the year 1797, he left Jefferies Square, and took up his residence at 12, St. Mary Axe, in the house which had been occupied for a long time by Mr. Cline, who, on removing into Lincoln's-Inn-Fields, strongly advised his young colleague to take possession of his late residence. At this time Astley Cooper being no longer desirous of retaining the office of demonstrator at St. Thomas's, and having a high opinion of Mr. Saunders, afterwards so eminent as an ophthalmic surgeon, he got that gentleman appointed in his stead. In 1798 Astley Cooper sustained a severe concussion of the brain, in consequence of a fall from his horse, and was confined to his room for five or six weeks. In the course of this illness a conversation occurred between Cooper and Cline, who was in attendance on him, which exemplifies the professional enthusiasm of the one and the philosophic *sang froid* of the other. Cooper, in the full persuasion that he should die, was lamenting the approaching event, not so much on his own account, as because it would arrest a train of professional inquiry in which he was then engaged, and which he thought would prove of the highest public benefit. "Make yourself quite easy, my friend," rejoined Cline, "the result of your disorder, whether fatal or otherwise, will not be thought of the least consequence by mankind."

About this time a grand discussion took place in the Physical Society of Guy's Hospital, between Astley Cooper and Abernethy. These distinguished surgeons held very different opinions on the treatment of injuries of the head; and the former invited the latter, by a sort of challenge, to a public discussion. An evening was accordingly fixed, and the theatre was crowded to the ceiling by the pupils of the rival professors. The debate was sustained with great spirit by both champions and their adherents; and, having been twice adjourned, terminated as arguments generally do, namely, by each party being more than ever convinced of the truth of their own opinion.

In the same year Astley Cooper became joint editor, with Drs. Haighton and Babington, of "Medical Records and Researches," which were the transactions of a private society connected with St. Thomas's hospital. Of thirteen papers contained in this volume, two were original essays by Cooper. He became a member of another society which was formed in the early part of the year 1800, which seems to have enjoyed a high reputation in its day, namely, the Edinburgh Club; so called because it consisted exclusively of gentlemen who had, at one period or other, studied at Edinburgh. Many eminent London practitioners were members of it, and all medical foreigners of distinction used to visit it. The discussions were carried on in a free and colloquial manner, without even the formality of any one taking the chair. The attention of the members, however, was entirely devoted to business; no cards or other species of amusement being allowed. A singular feature in the discipline of this society was, that the only stimulating beverage allowed to be drunk at its meetings was cold punch.

It need scarcely be intimated that a principal object of Cooper's ambition at this time was to become an hospital surgeon; and he was in all probability aware of his uncle's intention of retiring, in a short time, from Guy's. Mr. William Cooper, however, seems to have done nothing to favour the appointment of his nephew as his successor: on the contrary, he is supposed to have exerted his influence in behalf of another candidate. He seems to have been in some degree envious of the great fame and popularity of his nephew, and jealous also of the greater respect shown by the latter for the professional attainments of Mr. Cline, and his preference for St. Thomas's hospital. In the year

1800 Mr. W. Cooper resigned his office, and Astley Cooper started as a candidate, being opposed by three others,—Mr. Buckle, an army-surgeon; Mr. Whitfield, a brother of the apothecary of St. Thomas's hospital; and Mr. Norris, who, though entirely unconnected with the hospital, was Cooper's most formidable opponent, owing to the strong and influential exertions of Mr. Warner in his favour.

Astley Cooper stood on the strong ground of his own merits, and his services to the institution. The treasurer seems to have regarded him as professionally best fitted for the office; and he had on several occasions intimated with reference to this subject, the gratification he should derive from a change in Cooper's political opinions and associates: what these had to do with his qualifications as surgeon to Guy's hospital must have been more apparent to the treasurer than it is to us. On Cooper's political tenets, however, which, as we have seen, were sufficiently extravagant, his opponents rested their chief hopes of success; and during the canvass, the treasurer received an anonymous note deprecating, in the strongest terms, the election of a man holding such opinions. Mr. Harrison was much embarrassed as to the course which he ought to pursue, and, sending for Astley Cooper, explained to him the awkward position in which he had placed himself and his friends by his peculiar political relations.

We are sorry that our limits will not permit us to subjoin Cooper's answer in his own words, as communicated by Mr. Harrison to Mr. Bransby Cooper. The substance of it is that, in a late conversation between Mr. Colman and himself, they had come to the mutual conclusion that their political views were at once subversive of their peace of mind, and injurious to their success in life, and hence that they had resolved to discard them. The real moral philosophy of this change of sentiment we leave to the investigation of those who are profound in ethics; but, from the fact of no such abjuration having been made in Mr. Cooper's conversation with the treasurer a short time before, we surmise that this remarkable mutation was in some mysterious manner connected with the event of the pending election, and think on the whole that the less that is said about it the better.

As soon as this change in Cooper's political opinions was made known, and he had obtained the sanction of the treasurer, the tide immediately turned in his favour, and he was elected in October, 1800.

A short time previous to this event, Mr. Travers had been articled to him as a pupil, and resided in his house for some years. Mr. B. Cooper has inserted in his memoir a letter from Mr. Travers, illustrative of the character and habits of Astley Cooper at the period of his early acquaintance with him. This is on the whole the best written letter in the book.

Just prior also to his appointment to Guy's, Mr. Cooper had an accession to his household in his god-son Astley, the present Baronet and brother of Bransby Cooper. He had been prevented by the accident before referred to from attending the christening of the child, and he now saw him for the first time while on a visit to his father, the Rev. Lovick Cooper, who was resident at Yarmouth. He took a fancy to him, and, with his father's consent adopted him. On his return to London he brought the child with him, and presented him to his wife who, ever after bestowed upon him the care of a mother.

A domestic occurrence of no small importance to Astley Cooper happened this year, in the arrival of his celebrated servant Charles Osbaldiston, whose inconveniently long name was soon abbreviated into that of Balderson, which has been retained by him ever since.

By a rare combination of fidelity, shrewdness, and finesse, this person identified his master's interest with his own, and improved every opportunity with so much zeal and sagacity that he contributed no little to extend Mr. Cooper's practice, while he eventually raised himself to a substantial and respectable position in society. Mr. B. Cooper says he has heard him boast that, during the twenty-six years he was Sir Astley's professional servant, he never let but one case escape him in which it was possible to get his master called in.

A less laudable occupation of Mr. Balderson was that of procuring dogs for his master's experiments, and here the humanity either of master or man does not show to much advantage. We read with infinite delight of the escape of a friendly and confiding dog from the clutches of Michael the coachman. Sir Astley's relations to the canine species seem to have been by no means amiable, for we find him during his pupilage perpetrating, in conjunction with Mr. Holland, a most remorseless and inhospitable canicide on a poor destitute cur who followed them home in the hope of protection and kindness.

In reading the account of these way-layings and kidnappings of unhappy dogs, we are almost tempted to wish, for the sake of their tormentors, some of them had proved to be of the kith and kin of that tremendous "Pudel," who was trapped in the study of another great maker of experiments, Dr. Faust:

> Aber was muss ich sehen!
> Wie wird mein Pudel lang und breit!
> Er hebt sich mit Gewalt,
> Das ist nicht ein Hundes Gestalt;
> Welch ein Gespenst bracht' ich in's Haus;
> Schon sieht er wie ein Nilpferd aus,
> Mit feurigen Augen, schrecklichem Gebiss.

In furtherance of his anatomical pursuits, Mr. Cooper made an arrangement with the people connected with the menagerie at the Tower, to send to his house the bodies of all their dead animals. He also contracted with Mr. Halls, a stuffer of birds and other animals in the City Road, to purchase all his carcases with the exception of the skin. His supplies did not end here. All the fish and poultry markets in the city were laid under contribution, and Charles was a constant frequenter of Billingsgate, for the purpose of selecting such productions, as seemed better adapted to the scalpel than to the palate. All such specimens were put aside by the fishmongers for Astley Cooper, and if Charles happened not to make his appearance for a day or two, were not unfrequently sent to St. Mary Axe.

Astley Cooper's pursuits necessarily involved him in extensive transactions with the resurrection men of his day, and Mr. B. Cooper has thought it expedient to give not only a detailed history of these transactions, but also biographical sketches of all the principal gentlemen engaged in the reputable branch of commerce alluded to. Now this we think highly injudicious. We do not mean to deny that a very entertaining book might be written on the lives and fortunes of resurrection men; but we object to their introduction in the work before us, as being just as much out of place as would be the lives of the persons whose bodies were exhumed.

Had this been a scientific and professional life of Sir Astley Cooper, its chief interest would commence at the period at which we have now arrived; but as it is merely a private life, we find less incident, and consequently less to attract attention now than previously, and Mr. B. Cooper's second volume will demand a less detailed notice than the first.

We now find Astley Cooper fairly settled in life, occupying a very distinguished place in his profession, and regularly established in practice. In the year 1800, he laid before the Royal Society his views with respect to the influence on audition, of perforation of the membrana tympani, and, in the following year, a second paper entitled "Further observations on the effects which take place from the destruction of the membrana tympani; with an account of an operation for the removal of a particular species of deafness." Of the nature of this operation our readers are doubtless aware, and we need not dwell upon it, since Mr. Cooper's first case was the only one which was permanently successful; and although the operation has been frequently repeated with alleged success both in this country and on the continent, the fact of its having fallen into complete disuse seems to indicate its general inefficiency. For these two papers, however, the Royal Society awarded to Astley Cooper the Copleian medal, which is considered the highest honour they have to bestow, and which, fifteen years before had been awarded to John Hunter for certain researches in comparative anatomy.

In February 1805, Astley Cooper was elected a Fellow of the Royal Society. In the course of the same year he cooperated actively with Drs. Marcet and Yelloly in the formation of the Medico-chirurgical Society, which at first consisted chiefly of members who had seceded from the Medical Society in Bolt court, on account of a quarrel regarding the presidency. In the first volume of the Transactions of the Medico-chirurgical Society is an account of an operation for carotid aneurism by Astley Cooper, who had the honour of being the first surgeon who applied a ligature to the artery as a means of curing that disease. The patient died, but the circumstances of the case showed the applicability of the operation, and warranted future trials. A few years after he had the gratification of laying before the same society a case in which the operation was perfectly successful. He continued for some years occasionally to furnish papers to the society, and was also engaged in the preparation of his magnificent work on Hernia, to which disease he had himself been subject from a very early period of life.

Early in the year 1806, Mr. Cooper left St. Mary Axe, and moved into Broad street.

His work on Hernia appeared in two parts, the first in 1804 and the second in 1807. It at once established its author's reputation as an anatomist and surgeon of the first order. It was far, however, from being a source of pecuniary emolument to him. The plates accompanying it were got up in a needlessly expensive style, and when every copy of the work had been disposed of, Cooper found himself a loser by it to the amount of more than a thousand pounds. During the progress of this undertaking, he met with the zealous cooperation of his professional friends, who did not fail to supply him with all the materials relating to his subject which their own practice afforded. This work extended the fame of Astley Cooper all over the kingdom, and caused a great accession to his consulting practice. Shortly after its appearance, it was the occasion of an interview between himself and Mr. Hey of Leeds, whose name was also intimately associated with the subject of hernia. Cooper's views respecting a certain point in the anatomy of femoral hernia differed so essentially from those of Mr. Hey, that the latter commenced a correspondence on the subject, in consequence of which it was arranged that Mr. Hey should come to town, and that they should institute a conjoint examination of the point at issue. A day being appointed, Mr. Hey called in Broad street in the afternoon, and after he had inspected Cooper's preparations, they commenced their investigations on a subject which had been procured for the purpose. So interested were both in their occupation, that they continued it almost without intermission till between two and three o'clock in the morning, to the great discontent of Charles, who was candle-bearer on the occasion. The inquiry, however, terminated to the perfect satisfaction of the parties more immediately interested, and Mr. Hey left town fully convinced of the accuracy of Cooper's description.

Astley Cooper having recommended that his nephew Bransby, his present biographer, should be brought up to the profession of surgery, as that in which he should have most opportunity of being of service to him, this advice was acted upon, and young Bransby was placed under the care of Mr. Colman, one of the surgeons of the Norwich hospital, in order that he might acquire the rudiments of surgery and pharmacy previously to entering as a pupil at one of the London hospitals. Astley Cooper still continued to pay his visits to Norfolk, during one of which an incident occurred which was very characteristic of that unobtrusive kindness towards his professional brethren which made him so deservedly beloved by them. The reader will find it in Vol. ii, p. 67.

Astley Cooper was resolved not to sacrifice his nephew's true interests to any feeling of personal kindness. He told him plainly that he need not expect his support in the profession unless he deserved it; and Bransby having come to town some time before the commencement of the winter session, according to his account to "acquire a knowledge of the preliminary studies incident to a student's occupations in London," but in reality because he was tired of staying at Norwich, Astley Cooper entirely disagreed with his junior relative as to the necessity of such preliminary measures, which he perceived to mean simply a few weeks' idleness, and accordingly he started Bransby back to Norfolk the same evening, giving him a number of cases to transcribe for him during the vacant period before the sessions commenced. When the time arrived for his return his uncle sent him to live with Mr. Hodgson, now of Birmingham, and celebrated for his work on Diseases of the Arteries and Veins.

At this time Astley Cooper was lecturing on surgery assisted by Mr. Henry Cline, while Mr. Joseph Henry Green, Mr. Cline's nephew, held the office of demonstrator. Cooper's surgical class was larger at this time than any other in Great Britain, and perhaps the largest that he himself ever had. His practice too was now immense, and his time was most fully and laboriously occupied.

Mr. B. Cooper gives an entertaining account of his morning consultations, and of the dexterous manner in which Charles managed the concourse of patients. (Vol. ii, pp. 72-7.) The occupation of his time in hospital and private practice, as well as the great extent of his scientific pursuits obliged Mr. Cooper to engage persons to assist him in his researches. In the anatomical department he employed Mr. Lewis, who was the only person except himself who was allowed to enter his private dissecting rooms which were over the stables at the back of his house, and were fitted up with every convenience. Here Mr. Lewis usually commenced his day's labour at six in the morning, and was frequently occupied, with certain interruptions, till eleven at night. Early as was the hour of his arrival, he generally found Astley Cooper already at work; but, if not, there was always something on the table ready for him to proceed with, if the task on which he had been previously engaged had been completed over night. To these rooms were brought most of the diseased parts removed by operation or in post-mortem examinations in Mr. Cooper's hospital or private practice. Mr. Cooper was indefatigable in his exertions to eradicate the natural though injurious prejudice against necroscopic inquiries. When Mr. Lewis became connected with him, the task of obtaining permission for such investigations devolved on him, and so experienced did he at length become in the best mode of attaining his object, that from the time he was first employed by Mr. Cooper till he entered on private practice, he did not fail in more than six cases. For anatomical delineations, Astley Cooper first engaged an artist named Kirtland, who possessed good abilities, as evinced by the fact that many of the engravings in the work on Hernia were executed by him. He fell, however, into drunken and irregular habits, and Cooper was obliged to dismiss him. His place was supplied by Mr. Thomson, a miniature painter, whose talents had never been duly appreciated, and who was in poor circumstances. Mr. Cooper sent him to St. Thomas's to attend the anatomical lectures and to dissect, and he was thus rendered an efficient artist for the subjects on which his services were required. This gentleman in the course of his pursuits imbibed a considerable knowledge of surgery, and devoted himself particularly to the study of the eye. He ultimately left England for the West Indies, with a view of practising there as an ophthalmic surgeon, but died on the voyage. Mr. Cooper does not appear to have regularly engaged any other artist after Mr. Thomson left him; subsequently, however, to his removal from Broad street he secured the permanent assistance of Mr. Canton. In the formation of casts and models he had Mr. Lewis instructed by an artist named Schianelli, and he became very useful to him in this department. If in the course of the morning a patient called having a disease of any interest, to which this mode of copying might be applied, Mr. Lewis, having obtained his consent, would in a short time make a model of the diseased parts. Requests of this nature were very seldom refused by patients. Lewis appears to have been a man of great industry and varied capacity, for, in addition to his other occupations, he acted as Mr. Cooper's amanuensis.

In the manner above described, Astley Cooper's collection, which, when he first went to Broad street, consisted of only twenty preparations, soon became very extensive and valuable. In the winter of 1808 or 1809, he met with a slight accident; walking in the evening along Cannon street he slipped from the curb-stone and fell, and one of his feet becoming fixed in some ice, the fibula was in consequence fractured. This accident, however, did not long detain him from his practice. While in Broad street, Mr. Cooper gave frequent soirées, which were attended chiefly by the physicians and general practitioners whom he was in the habit of meeting, or his dressers and favorite pupils at the hospital. He also made it a rule, once a week, to invite certain of these professional acquaintances to dine with him. He was a member of several clubs, the meetings of which he frequently attended; as that of the Athletæ, the Kent Medical Club, and the Pow-wow. The Athletæ, who were embodied, as their name imports, for the cultivation of active exercises, consisted chiefly, though not entirely, of medical men. The late Dr. Babington, who greatly excelled in athletic feats, was one of their most active members. The Athletæ seem to have been very funny and good-humoured fellows, but we have not space to detail their recreations. The origin of the Pow-wow club is disputed, some referring it to John Hunter, and others to his intimate friend Dr. Patrick Russell. The word Pow-wow in some of the eastern languages signifies a meeting of conjurers, and appears to have been adopted by the club as a caprice. However these things may be, the Pow-wow seems to have been a source of much gratification to some of the most distinguished men in the profession. It was dissolved in 1830.

In the early part of the year 1806, Mr. Cooper evinced his acuteness in detecting the murderer of Mr. Blight of Deptford, whose death occasioned so great a sensation at the time. He was called in to Mr. Blight, whom he found in a hopeless condition, from a wound inflicted with a pistol by an unknown hand. Circumstances led him to suspect Mr. Patch, the partner of Mr. Blight, and on investigation of all the facts connected with the discharge of the pistol, he became convinced that it must have been fired by a person who was left-handed. On learning that Patch was left-handed, he entertained no further doubt on the subject, and on his return home said to his servant, in secrecy, "you will see, Charles, that Mr. Patch, the partner of Mr. Blight, has been his murderer." And so it turned out, though no one but Cooper entertained any suspicion of the truth until the inquest on the body of Mr. Blight, when, strong evidence appearing against Patch, he was committed for trial, and subsequently condemned and executed.

Some years afterwards, Astley Cooper displayed the quickness of his intuitive powers in the detection of another remarkable murderer. Mr. and Mrs. Thomson Bonar had their town-house in Broad-street, near that of Mr. Cooper, and were on intimate terms with him. One morning Mr. Cooper was horrified at the arrival of Nicholson, the servant of Mr. Bonar, who came on horseback from that gentleman's country residence at Chiselhurst, to say that Mr. Bonar had been murdered during the night, and that Mrs. Bonar was in a very dangerous state from the wounds which she also had received from the assassin. Mr. Cooper was at once convinced, by the appearance and manner of the messenger, that he was himself the murderer, and when setting off for Chiselhurst, he desired Mr. Frederick Tyrrell, then his pupil, to stay in town and look after Nicholson. The truth of his suspicions must be known to all our readers who can recollect the case.

At the period of which we are speaking, many, if not most, of the principal merchants in London had residences in the city. This had an immense influence on Astley Cooper's income; for although, perhaps, he might not see so many patients in a day as after his removal to Spring-Gardens, the remuneration he received was much greater. The merchants generally gave him a cheque instead of a fee, and no one wrote for less than five guineas, though had the payment been made in cash it would have been only two. When sent for out of town, he was also remunerated with extraordinary liberality. Mr. William Coles, of Mincing Lane, for years paid him £600 per annum for visits to his seat near Croydon. In the year 1813 he performed the operation for stone on Mr. Hyatt, a West India merchant, and received from him probably the largest fee ever given by a private individual for a surgical operation.

"The manner in which the fee was presented was not, perhaps, the least extraordinary part of the circumstance........His patient, who was sitting by the fire, took off his night-cap and jocularly threw it at him, saying at the same time, 'there, young man, put that into your pocket.' Mr. Cooper, guessing at the contents of the missile, took from it a piece of paper, and, tossing the cap back to his patient, took his leave. The paper was a cheque for a thousand guineas."

While Astley Cooper maintained these agreeable and profitable relations to the public, he was also particularly happy in the terms on which he stood with his own profession.

"It appeared," says his biographer, "as if he had by some magic gained the confidence of every medical practitioner who had access to him; and this insured an extension of his fame over a very large portion of England. This influence did not arise from his published works, nor from his being a lecturer, nor indeed from any public situation which he held, although each of these circumstances had its share in producing the result; but it seemed to originate more from his innate love of his profession, his extreme zeal in all that concerned it, and his honest desire, as well as great power, to communicate his knowledge to another, without, at the same time, exposing the ignorance of his listener on the subject even to himself." (Vol. ii. pp. 162-3).

All this is strictly just, and in no respect more so than in reference to his power of enlightening other practitioners on points of which they were ignorant, without, in the smallest degree, wounding their self-love. This was one of the most amiable as well as useful traits in the professional character of Sir Astley Cooper.

At this time the gullibility of the public was severely taxed by a most amusing set of scamps, termed by those who understood their manœuvres, the "Ashley Cooper Set." They were a junta of advertising quacks, who held solemn sessions in robes and wigs, presided over by a personage styled "Doctor Ashley Cooper." We have not space to record their proceedings; but the reader will find in Mr. B. Cooper's work, an amusing account of these, as well as of some other impostors who founded their schemes on the fame of Astley Cooper.

In the year 1811 Mr. Cooper purchased the estate of Gadesbridge, at Hemel Hempsted, in which he does not appear to have made a very good bargain; though both he and his wife were pleased with their new acquisition.

In May, 1813, Mr. Cooper was appointed Professor of Comparative Anatomy to the College of Surgeons. The immense labour entailed by this appointment would have disinclined him from accepting it; but as there was no one at that time connected with the college willing, or perhaps very competent, to undertake the task, he considered it a duty on his own part to do so. On accepting the appointment, Mr. Cooper devoted himself with the utmost zeal to the preparation of his lectures. His engagements would not allow him to study the works of the various authors who had written on the subject, but he gave up all his leisure time to the practical investigation of it by dissection, and even curtailed the period of his nightly rest, brief as that already was. Mr. Lewis having in the preceding year entered upon practice, Mr. Cooper employed Mr. Parmenter in the same capacity, and found him a most efficient assistant. While these lectures were in course of delivery, the death of Mrs. Parmenter, the adopted daughter of Astley Cooper, so disturbed his mind, that he felt himself unequal to sustain such an accumulation of labour, and an abrupt termination was put to the lectures.

In the same year he removed from Broad street to New street, Spring Gardens. One motive which induced him to take this step was, that he might be in the near vicinity of certain connexions which he was forming about the court, to be attached to which had now become an object of his ambition. But his chief motive was to escape from the excessive fatigue of his immense city practice, the extent of which may be inferred from the amount of his annual income, which, in the year 1815, exceeded twenty-one thousand pounds.* His multifarious labours were beginning to tell on his health. His spirits and his appetite did not fail; but, from privation of exercise, he had become corpulent, and his naturally ruddy complexion assumed now and then a bluish tint when he used exertion. He was subject also to attacks of vertigo; and one morning when the Duke of Manchester had called to consult him, he was seized with one of these attacks and fell prostrate. His subsequent demeanour on this occasion was very characteristic of the care and caution so conspicuous in all his professional doings. On recoveriug himself, his first words conveyed an earnest request to the Duke that he would never speak to any one of what he had just witnessed, a request with which his Grace strictly complied. This circumstance was so closely concealed during the life of Sir Astley, as to have been known only to the Duke of Manchester, Lady Cooper, Mr. Bransby Cooper, and the trusty servant Charles.

In May, 1816, according to our biographer, Astley Cooper performed his celebrated operation of placing a ligature on the aorta. But Mr. B. Cooper is here at fault in his chronology: the operation in question was performed on the 25th of June, 1817. This was, beyond all comparison, the boldest enterprise in the annals of operative surgery. When it became known to the profession it excited general amazement, and some did not hesitate publicly to express their doubt whether any circumstances of danger could justify so fearful an expedient. It would be dissonant from the character of the present article to discuss the merits of this operation, which is the less needful because it is now admitted to have

* This sum includes all monies paid into his banker's hands on his account; his actual *fees* for one year *could* not have amounted to this vast sum.

been wisely and advisedly undertaken, and to have been so borne out by the circumstances of that case, and of others in which the operation has been repeated, as to justify its repetition and afford a hope of eventual success.

In the year 1818, Mr. Cooper and his distinguished pupil, Mr. Travers, who was now one of the surgeons of St. Thomas's Hospital, published conjointly the first volume of their Surgical Essays. The very favorable reception of this led to the publication of a second part, as soon as circumstances would permit; the work nevertheless was not continued, owing, as Mr. B. Cooper thinks, to the conviction of Astley Cooper that the publication of these essays in volumes appearing at distant intervals would be less useful than the separate exposition in complete treatises of the important subjects to which they related.

In the year 1820, Astley Cooper lost a much valued friend by the death of Dr. Marcet, of whom a biographical sketch is given. His history is curious, but we have not space to advert to it.

From this period Mr. Cooper was in frequent attendance on Lord Liverpool until the death of that nobleman. They were on terms of intimate friendship, and appear to have entertained a very high opinion of each other. Mr. B. Cooper has given some anecdotes of his lordship from the papers of Sir Astley, which are worthy of perusal.

In the same year, 1820, Mr. Cooper was called into attendance on George the Fourth, although, at that time, he held no official situation about his majesty's person. He seems to have been sent for merely on the strength of his great professional reputation, which had reached the ears of the king. In the spring of 1821 he removed a steatomatous tumour from the head of the royal patient, in the presence of Sir H. Halford, Sir M. Tierney, Sir Everard Home, Mr. Cline, and Sir B. Brodie. The length to which this article has already run, prevents us from noticing the details of his attendance on the king, and the anecdotes connected with it: most of these, indeed, have come repeatedly before the public through other channels.

The observations found in Sir Astley's notes relative to George the Fourth are extremely flattering to that monarch, but give far too high an estimate of his knowledge and attainments. They seem written too much in the spirit of a courtier, and rather indicate that Sir Astley had passed into an extreme of political sentiment, opposite to that into which he had fallen in his youth.

It might be thought that the strong disinclination of Mr. Cooper to undertake the operation on the king, and his attempt to devolve it on another, savour of timidity, selfishness, and littleness of mind. The following is his own account of his feelings:

"I called upon Lord Liverpool, and requested him to persuade the king to let Home do the operation, as that was the usual etiquette, he being sergeant-surgeon. Lord Liverpool said that it was very difficult to interfere respecting the choice of a medical man. I was very averse from doing it; I had always been successful, and I saw that the operation, if it were followed by erysipelas, would destroy all my happiness and blast my reputation.........Soon afterwards Sir Henry Halford was called out of the room, and almost immediately returning, said to me, 'you are to do the operation.' I was thunderstruck, and felt giddy at the idea of my fate hanging upon such an event." (Vol. ii. p. 229.)

Sir Astley has here omitted to mention what abundantly exculpates him: he has however mentioned it in another place. He was still subject to those attacks of vertigo, one of which overwhelmed him in the presence of the Duke of Manchester, and he observes, "this state of my nerves made me dread the operation upon George the Fourth, as I could not help fearing that I might be similarly seized at that moment."

It was not the moral man that was to blame; for nothing annihilates confidence in oneself so completely as the apprehension of any sudden seizure which suspends the command over one's faculties: moral resolution is based on the full consciousness of such command, and when this is no longer felt the firmest resolution will waver.

Shortly after the operation, the king made Mr. Cooper a Baronet, and, in compliance with his request, entailed the title upon his nephew and adopted son, Astley.

Sir Astley afterwards received as a present from his majesty a beautiful epergne, which cost five hundred guineas, and for which the king gave the plan himself.

Two days after Astley Cooper's elevation to the baronetcy, his heir was married to Miss Rickford, the only child of William Rickford, Esq. M.P. for Aylesbury. This marriage gave Sir Astley the greatest satisfaction, and he always showed the kindness of a parent to the young couple.

In the year 1822, Sir Astley Cooper was elected one of the Court of Examiners of the College of Surgeons. His conduct in this capacity was characterized by remarkable kindness towards the candidates.

"This was not only evinced by his expressions of encouragement, but equally so by his kind of examination, for he never asked catch-questions, or made abstract inquiries; but invariably dwelt upon practical matters, worded in simple and straightforward language. It was always a most painful task to him when it became his duty, as president, to reject candidates, and he mingled on these occasions with his terms of disapprobation, such friendly and even paternal advice, that he always succeeded in lessening the pangs of disappointment, and has been known to be the means of thus changing a young man's habits from idleness and dissipation to those of industry and professional study. ..........I have frequently known him, when pupils have been rejected at the College of Surgeons, write, unsolicited, to explain to their parents the circumstances under which they had been sent back, so as to mitigate their regret, and prove to them that a few weeks' more study would remove the obstacle which had prevented the admission of the young man as a member of the college." (Vol. ii. pp. 245-6.)

In the same year he brought out his great work on Dislocations and Fractures of the Joints, a considerable portion of the substance of which had already appeared in the Surgical Essays. It is needless to say that this work invested the name of Astley Cooper with new honours, and was executed in the masterly style which is common to all his surgical writings. Towards the latter end of the year 1824, his attacks of giddiness became more frequent, and were occasionally attended with difficulty of breathing, from which symptoms, however, he was invariably relieved by a few days' retirement into the country. Being somewhat alarmed at the frequent recurrence of these attacks, and attributing them in a great measure to the exertion of lecturing, he limited himself, from the commencement of this season, to the delivery of the surgical lectures. After Christmas, however, he resolved to give up lecturing entirely, and sent in his resignation to a general meeting of the governors of the hospital in January, 1825. For some time before his resignation Mr. Key had taken a part in the surgical lectures, and Mr. Bransby Cooper had officiated instead of Sir Astley in the anatomical course. As he was satisfied with the manner in which these gentlemen had fulfilled their duties, he wished not to retire without an understanding that they would be appointed as his successors, and it was under the conviction that this condition had been acceded to by all parties that he tendered his resignation. Contrary to his expectations, however, Mr. South was appointed to the anatomical chair, and a feud hence originated which led to the establishment of a school of medicine at Guy's hospital, and the separation of what had hitherto been called the United Borough Hospitals. In connexion with these events, a duel had like to have taken place between Sir Astley and Dr. Cholmondely. The doctor, however, having put himself in the hands of Mr. Power, to do as he thought right for him, and that gentleman being of opinion that he was in the wrong, he made a public apology, and thus the affair ended.

On the establishment of the new school at Guy's, Sir A. Cooper exerted himself in the formation of a museum adequate to illustrate the lectures; for his former preparations, which had been deposited at St. Thomas's, were lost to him in consequence of his secession from that school. As soon as he was established in New Street, he began to accumulate similar preparations, which were immediately taken to Guy's, and being added to those already placed there by Dr. Haighton and others, formed the nucleus of what is now one of the most valuable museums in Europe.

The farming and sporting occupations of Sir Astley at Gadesbridge, of which a sufficiently entertaining account is given, we must entirely pass over. Like most gentlemen farmers, he derived more pleasure than profit from his pursuits.

In 1824, Sir Astley performed the operation of lithotomy on the Vice-chancellor, Sir John Leach, whose fortitude under a severe and protracted operation excited general admiration.

In October, 1826, he was called into consultation on the case of the Duke of York, and continued to attend him till his death. Various anecdotes of his Royal Highness are given from Sir Astley's note-book.

In January, 1827, Mr. Cline died, and Sir Astley was naturally much affected at the loss of his old friend and preceptor, the more so because circumstances connected with the separation of Guy's and St. Thomas's hospitals had led to a partial estrangement between them.

In 1827, Sir Astley Cooper was elected President of the Royal College of Surgeons; an honour which was again conferred on him in 1836. In the month of June, in the same year, he lost his wife, who died at Gadesbridge, of erysipelas of the head. The grief occasioned by this event increased the frequency of Sir Astley's attacks of vertigo, and he was often obliged to protract his visits to the country longer than he had proposed. This continual interruption of his professional pursuits so annoyed him, that, in September of this year, he determined to retire from practice, and give himself up to a country life on his estate.

For a short time he was much gratified by the change, and delighted at the novelty of having his time entirely at his command; but, as might have been anticipated, he soon began to experience an ennui to which he had hitherto been a stranger, and felt the want of that excitement which long habits of professional activity had rendered necessary to his happiness. During his retirement in the country he had an attack of intermittent fever, which had nearly proved fatal. On his recovery he wisely resolved not to allow the fear of being laughed at for so sudden a change in his plans to deter him from resuming the practice of his profession; and, on the re-establishment of his health, he returned to London, after an absence of about six months. At first he took lodgings, intending merely to see a few of his old patients three or four days in the week. His return, however, was no sooner known to the public than patients crowded in upon him; and, his health being restored, he resumed practice with little less than his former zest. He soon afterwards purchased the lease of a house in Dover street, but not finding this tenement suited to him, he disposed of it at a loss, and took the house in Conduit street, in which he remained till his death. His old servant Charles, whom he had placed on a farm called Warner's End, did not return to him.

In July, 1828, Sir Astley again entered into the married state, espousing Miss C. Jones, with whose family he had been acquainted from the earliest period of his residence in London.

In the same month he was appointed sergeant-surgeon to the king.

In 1829, he published the first part of his admirable Illustration of the Diseases of the Breast. In 1830 he was elected vice-president of the Royal Society, being already a member of the council.

Towards the latter end of the summer he made, in company with his wife and another lady, a tour of considerable extent on the continent. Mr. B. Cooper has published Sir Astley's notes of this journey, but, as we think, very injudiciously, as they contain nothing but common-places. In the autumn of 1831, he made a tour over the southern and south-western parts of England. On his way home he purchased at Lyme Regis, of the well-known Mary Anning, a very fine specimen of the ichthyosaurus, a drawing of which afterwards appeared in Dr. Buckland's Bridgewater Treatise.

On the accession of King William the Fourth, his appointment as sergeant-surgeon was confirmed. He had been brought into personal intercourse with the king, when Duke of Clarence, as early as the year 1818, when he attended his Royal Highness's son, then Captain Fitzclarence, who had received a severe compound fracture of the leg, in consequence of a fall from his horse, and who, ever after, retained a grateful sense of Sir Astley's attention to him.

Early in the following year Sir Astley published his work on the Anatomy of the Thymus Gland, which he dedicated to Dr. Babington, whom he very highly esteemed, in common with all his medical brethren, and who shortly after, fell a victim to the influenza then prevalent.

At this time Astley Cooper was elected a member of the Institute of France, and received from the King the rank of officer of the Royal Order of the Legion of Honour. Shortly afterwards he was elected a corresponding member of the Royal Academy of Sciences, a place having become vacant in the section of medicine and surgery, in consequence of the death of M. Delpech.

In June, 1834, on the occasion of the installation of the Duke of Wellington at Oxford, Sir Astley Cooper received from that university the honorary degree of Doctor of Civil Law.

In this year he made another tour with Lady Cooper, and some members of her family, over a considerable part of Germany, and through Switzerland into France. The acquisition of professional knowledge was still one of his principal objects. There was not an hospital in his route that he did not visit; and he not unfrequently made a detour for the express purpose of examining some public museum or private collection illustrative of his favorite pursuits.

In the year 1835, Sir Astley was elected an honorary fellow of the Royal College of Surgeons of Edinburgh, and soon afterwards determined to make a tour to Scotland; he was, however, prevented by various engagements, from accomplishing his object till 1837, in August of which year he arrived in Edinburgh. On the 5th of September a dinner was given him by the College of Surgeons, when sixty gentlemen, Fellows of the College, met to welcome him. On his health being drunk, Sir Astley returned thanks in a long speech, in the course of which he took a review of his past life, comparing his position at that time with his situation and prospects when he had been among them as a student, and stating the circumstances to which he attributed his success.

On the morning of the same day, the degree of LL.D. had been conferred upon him by the university. On the same day also, at a meeting of the magistrates and council, the freedom of the city was voted to Sir Astley, and on the following morning a deputation, consisting of the Lord Provost and a large number of the council, accompanied by many members of the university, arrived at the hotel at which he was staying, and presented him with the customary documents. In the afternoon he set out on a tour to the highlands. On reaching Carlisle in his homeward route, he writes,

"This has been a delightful and flattering tour in Scotland; delightful from the scenery, the variety of the prospects, the goodness of the roads, and is flattering, from the great civility we received in Edinburgh and Glasgow, and, indeed, wherever we went. It was the reward of a life spent in the industrious pursuit of a profession."

Three years after this tour he was proposed with two other candidates, the Duke of Wellington and the Marquess of Breadalbane, for the office of Lord Rector of the University of Glasgow. The Marquess was elected.

In September, 1835, Mr. Bransby Cooper was alarmed at a false report of Sir Astley's sudden death in the Isle of Wight, which he heard casually in a stage-coach. Having ascertained the inaccuracy of the report, he wrote to his uncle to tell him of the apprehension he had been under. Sir Astley returned an answer which begins as follows:

"My dear Bransby,—It is with much self-gratification that I assure you that I am not *dead,* and that the only fit I have had is a fit of hunger, to which disease I have been extremely liable ever since I was born. Indeed, it is my full intention to practise my profession for the next thirteen years; after that time to retire for twenty, and then be at God's disposal for as many more as he pleases. I suppose the false report has arisen out of Brodie's accident, whom I saw at Newport very well; but the injury he met with was severe." (Vol. ii. p. 448.)

In the year 1840, Sir Astley published his last work, On the Anatomy of the Breast, a work of transcendent merit, and truly astonishing, when we consider that the author, at the time of its appearance, was upwards of seventy years of age, and that its materials were chiefly derived from researches undertaken in the latter years of his life. During the progress of this work he found time to contribute several papers to the Guy's Hospital Reports, the first volume of which appeared in 1836. These papers contain the results of dissections, experiments, physiological inquiries on various subjects, and numerous surgical observations, and tended greatly to promote the success of the work.

The tone of the letter above quoted does not indicate any consciousness of a failure in the powers of his constitution. He had, however, continued subject to his attacks of vertigo, which latterly had been resolved by fits of gout; and towards the end of 1840, he became afflicted with extreme difficulty of breathing, greatly augmented by any ascent. A short time before Christmas he performed an important operation upon Lady ——. Before he would undertake it, he sent Balderson* to ascertain the number of stairs which led to her bedroom, saying "if there are more than lead up to my own bedroom, I leave it to you to manage that the lady shall be moved to an apartment in the lower story, otherwise I shall not attempt to operate."

At Christmas, Sir Astley and Lady Cooper went to the Rev. Mr. Board's, at Westerham, to pass a fortnight, as they had been in the habit of doing for several years. That gentleman was at once struck with the failure of Sir Astley's health and spirits. When the period fixed for his return to town arrived, he was too ill to leave the country; but anxious to conceal from his friends the real cause of his delay, and the serious nature of his illness, he wrote to town merely saying that he intended to prolong his visit at Westerham for another week. Mr. Bransby Cooper, however, was quite convinced that nothing but serious indisposition could detain him so long from his occupations in Conduit street, and he therefore wrote entreating him to return to town and place himself under medical treatment. A few days afterwards Sir Astley arrived in Conduit street.

"I called and saw him," says Mr. B. Cooper, "and never was I so shocked as when I witnessed the change which one short month had worked upon a frame which had always hitherto indicated health and vigour, and been graced by a countenance expressive at once of intellect and happiness. On entering the room I found him sitting with his head bent upon his chest, and, instead of greeting me in his wonted lively and affectionate manner, he scarcely moved; but, with a half-extended hand and melancholy expression, watched narrowly what impression his altered appearance excited in my mind. I could not conceal my emotion: he observed it, and pressed my hand. The interview was too painful to me to sustain for any lengthened period, and I left the house fully convinced that my poor uncle's days were drawing rapidly to their close. (Vol. ii. p. 452.)

He died on the 12th of February, 1841, in the seventy-third year of his age. For some days previous to his death, he had frequent attacks of delirium, but in the intervals he affectionately recognized his friends, and his frame of mind was that of religious resignation to the will of

* Who, we presume, was on a visit to his old master, as we read of his having quitted his service some years before.

God—for he was perfectly aware of his condition. He had been attended during his last illness, by Drs. Bright and Chambers, and Messrs. Key, Tyrrell, and Bransby Cooper. He derived great comfort from the spiritual ministrations of his nephew, the Rev. Beauchamp Cooper. The remains of Sir Astley Cooper were interred by his own desire, beneath the chapel of Guy's Hospital. A few days after the funeral, a meeting of the elder pupils of the hospital was held, for the purpose of erecting, by subscription, a tablet to his memory in the chapel. This design, however, was abandoned, and, in its stead, a bust, executed by Mr. Town, has been placed in the museum of the hospital. Another bust, to be executed by Mr. Behnes, is to be placed in the council-room of the College of Surgeons. The senior members of the profession at large met at the Freemasons' Tavern, and formed a committee to receive subscriptions for the purpose of erecting some monument to record the services rendered by Sir Astley Cooper to the public; they have since determined on placing a colossal statue of him in St. Paul's Cathedral. This work, which has been intrusted to Mr. Baily, is still in progress.

Sir Astley Cooper established a triennial prize of three hundred pounds for the best original essay on some medical subject, and determined the first six subjects himself. The competition for this prize is open to all members of the profession in this country, or any other, except the medical staff of Guy's and St. Thomas's hospitals, who are appointed the adjudicators of the prize.

The general character of Sir Astley Cooper will be sufficiently collected from the foregoing sketch of his life. We regret much that we are debarred from dilating on his professional character by the nature of the work under review. We may, however, transcribe Sir Astley's opinion of himself contained in some notes on the characters of the principal surgeons of the day:

"Sir Astley Cooper was a good anatomist, but never was a good operator where delicacy was required. He felt too much before he began ever to make a perfect operator. For the operation of cataract he was quite unfitted by nature. Quickness of perception was his forte, for he saw the nature of disease in an instant, and often gave offence by pouncing at once upon his opinion. The same faculty made his prognosis good. He was a good anatomist of morbid, as well as of natural, structure. He had an excellent and useful memory. In judgment he was very inferior to Mr. Cline in all the affairs of life, and hence he was continually walking upon a mine ready to explode under his feet. His imagination was vivid, and always ready to run away with him if he did not control it." (Vol. ii. p. 475.)

Sir Astley, however, here underrates his own powers as an operator. He was not a particularly neat operator, but he was self-possessed, dexterous, and *successful* in a degree seldom equalled. We think, also, he errs with respect to the extent of his imagination. We do not believe he had a particle of imagination in his composition. He probably mistook for it the vivacity of mind arising from his quick and vivid perceptions.

We must not conclude this article without some reference to the merits of Mr. B. Cooper's book. We here repeat the remark with which we set out, namely, that the plan and object of the work are essentially faulty. The author, with the best feelings and intentions, has done injustice to the memory of his illustrious relative by presenting to us the great Sir

Astley Cooper, divested of nine tenths of all that made him great, and, consequently, reduced to very moderate dimensions. We wish we could speak favorably of it in a literary point of view; but this is impossible. It would appear that the task of its composition had been imposed on the author by the will of his late uncle—a will so sacred to his affectionate heart, as to induce him to undertake a labour for which he was very imperfectly fitted by previous habits of study. Had he, indeed, undertaken the *professional* life, the want of which we so much regret, the result, we doubt not, would have been very different, as he would then not only have found himself perfectly at home in his own proper style of composition, but would have been amply qualified by his critical knowledge of all the subjects treated of. Entering abruptly and without preparation on the field of general literature, Mr. Cooper shows manifest unacquaintance with the ordinary modes of proceeding and conventional customs of the cultivators thereof, and even with the common weapons wielded by them. At the same time, it must be admitted that he brings with him a vigorous constitution of mind, and strong and ready mother-wit, which need only the training of experience and the study of good examples, to issue in works of a very different character from the present. Above all, Mr. Cooper evidently possesses the candour, the goodness of heart, the kindliness of nature, and the innate sense of honesty and honour, which form so important elements in the composition of a good writer, and without which, intellectual powers, however great, can never take a permanent hold on the sympathies of men. As it lies before us, however, a sense of justice compels us to say that the work is in many parts incorrectly and feebly written, and that the materials are heterogeneous and ill digested. If it had fallen to the lot of a Greek critic to review this book, its violations of the "unities of time, place, and action," would inevitably have been the death of him. In our analysis of it, we have been obliged now and then to transpose an event over a space of twenty years or so, in order to render the narrative in any degree consecutive. We must observe also, that some passages are in very bad taste, and are much too redolent of the hospital and the dissecting-room. For example, an anecdote is related of a trick practised upon a hair-dresser, by Astley Cooper and Mr. Colman, from which they derived infinite amusement; whereas we perceive in it much nastiness but no fun, and are sorry that it has been published.

With all its faults, however, the work is exceedingly entertaining, and contains a vast store of professional anecdote and gossip which is not to be found elsewhere. Some of the stories are well told, and there is a vein of good humour running through the volumes, which renders them pleasant to read. Although as honest critics, we cannot speak favorably of the literary character of Mr. B. Cooper's book, we nevertheless strongly recommend our readers to peruse it. We ourselves, in fact, having read it once for the purposes of criticism, intend to read it again for the sake of amusement.

